# Oral cancer awareness amongst hospital nursing staff: a pilot study

**DOI:** 10.1186/1472-6831-9-4

**Published:** 2009-01-28

**Authors:** Lachlan M Carter, Andrew T Harris, Vikram P Kavi, Sarah Johnson, Anastasios Kanatas

**Affiliations:** 1 Oral and Maxillofacial Surgery, Hull Royal Infirmary, Anlaby Road, Hull, HU3 2JZ, UK; 2 Cancer Research UK Training Fellow, Floor 6, Worsley Building, University of Leeds, Clarendon Way, Leeds, LS2 9LU, UK; 3 Oral and Maxillofacial Surgery, Oral and Facial Specialties Department, Pinderfields General Hospital, Aberford Road, Wakefield, WF1 4DG, UK

## Abstract

**Background:**

Oral cancer is as prevalent as cervical and testicular cancer in the United Kingdom. Nursing staff provide the oral health care for the patient population in hospital. Admission to hospital provides a 'window of opportunity' for oral cancer 'screening' via an oral health check during nursing clerking. This study aimed to investigate whether nursing staff are aware of risk factors for oral cancer, its clinical signs, and could therefore provide a 'screening' service for oral cancer.

**Method:**

Through the use of a questionnaire we assessed 121 nursing staff on oral health check behaviour and attitudes; their knowledge of risk factors for oral cancer; their understanding of common clinical signs of oral cancer; and their undergraduate and postgraduate training in oral health and oral cancer.

**Results:**

Over 80% thought oral health checks were important although only 49% performed this task regularly; approximately 70% identified smoking as a risk factor but less than 30% identified alcohol. Awareness of the clinical signs of oral cancer was low with 21% identifying white patches, 15% identifying ulceration and only 2% identifying red patches despite their malignant potential. Nurses within 3 years of qualification were significantly better at recognising risk factors for oral cancer than their colleagues, identifying a need for continuing postgraduate education on oral health and oral cancer. Sixty-one percent of nursing staff received oral healthcare as an undergraduate with 34 percent receiving postgraduate training.

**Conclusion:**

An oral health check upon admission to hospital provides an opportunity for nurses to 'screen' for oral diseases including oral cancer and allows nurses a greater role in total patient care. Nurses' awareness of oral cancer risk factors and clinical signs was, however, poor. This study highlights a need for improved education of nurses on oral cancer to make the oral health check on admission viable for oral cancer screening.

## Background

In the United Kingdom oral cancer is as prevalent as cervical and testicular cancer. The incidence is increasing particularly in Scotland and in younger patients [1-5]. In the United Kingdom there is a screening service for cervical cancer, however, no such campaign exists for oral cancer. There have been initiatives such as: Mouth Cancer Awareness Week (MCAW) and the West of Scotland Cancer Awareness Project (WoSCAP), to increase the general public understanding of this disease. However, the general population's awareness of these conditions even in light of such teaching events is still considered poor [6]. Oral cancer mainly affects individuals in the 6^th ^and 7^th ^decades of life with a history of smoking and, or alcohol consumption. Early recognition and referral is essential as less treatment is needed and cure rates and 'quality of life' are much better [7-10]. Previous studies have shown that oral cancer can be silent in symptomology with awareness of early signs being more beneficial in diagnosis [11]. Oral cancer has four cardinal signs which warrant further investigation. These are erythroplakia, leukoplakia, mixed (erythroleukoplakia), and ulceration. Of these the commonest presenting sign is ulceration. The preponderance of this disease is identified through general medical and dental care [12-14]. However, there is evidence to suggest general medical practitioner and general dental practitioner lack of awareness contributes to a delay in diagnosis [13]. There is also evidence that the elder generation do not seek dental care [15]. Nursing staff provide oral health care for the patient population in hospital; this is especially true of the vulnerable elderly cohorts who are more likely to suffer from oral cancer. Therefore, admission to hospital provides a 'window of opportunity' for 'screening' for this disease. It is paramount that nursing staff are aware of the risk factors and clinical signs of oral cancer. This study questioned hospital based nursing staff in the East Yorkshire region on their awareness of oral cancer, its risk factors and presenting signs.

## Method

### Participants

Ward based nurses, independent of gender or levels of experience at two East Yorkshire hospitals were recruited from various medical and surgical specialities. Their oral cancer awareness was assessed by means of a questionnaire. Nursing staff were approached directly by the researchers and asked to complete the questionnaire with the researchers present.

### Questionnaire

The questionnaire assessed; oral examination habits, knowledge of oral cancer; its clinical appearance, and risk factors. This questionnaire was based on previous work by one of the authors [16,17]. The questions used were mainly open-ended rather than closed questions, thus hoping to avoid bias and evaluate knowledge base. See appendix A for questionnaire. The results were analysed using ANOVA, student's t test and chi squared tests where appropriate.

### Ethical considerations

Ethical approval was sought from South Humber research ethics committee and deemed not necessary for this study (reference number: 05/Q1105/47).

## Results

Of the 123 nurses approached, 121 agreed to complete the questionnaire, producing a return rate of 98%. The distribution of nurses by specialty is shown in table 1. Surgical specialties included: General surgery, Maxillofacial surgery, Otorhinolaryngology, Neurosurgery, Orthopaedics, Paediatric surgery, Plastic surgery, Vascular surgery, Urology, and Gynaecology. Medical specialities included: Medicine for the Elderly, Gastroenterology, Haematology, General Medicine, Paediatrics, Respiratory medicine, Rheumatology, and Stroke medicine. The Critical Care group included nurses from Adult and Paediatric Intensive Care and High Dependency Units. Bank nurses were treated as a separate group as they may not have a specialist interest/practice. Seventy one nurses (58%) graduated from nursing school three or more years ago, 13 nurses (11%) were between one and three years post graduation and 14 nurses (12%) graduated less than one year ago. The remaining 23 nurses (19%) were auxiliary nurses. Seventy-four per cent of nurses questioned reported they were regular dental attendees.

**Table 1:** Distribution of nurses by specialty

	n	%
Accident and Emergency	5	4
Surgical specialties	43	36
Critical care	23	19
Medical specialties	43	36
Bank/pool nurses	7	5

Forty nine percent of nurses reported that they carried out an oral health check upon admission. One nurse reported that they sometimes carried out an oral health check and 49% reported that they did not perform an oral health check. An oral health check was performed by 30% of surgical nurses, 20% of accident and emergency nurses, 56% of critical care nurses, 64% of medical nurses, and by 71% of bank nurses. The oral health check involved asking questions regarding the oral health care needs of the patient, and any oral symptoms in addition to examination of the patient's mouth. Over 80% of nurses agreed that it is important to examine a patient's mouth on admission; however, only 49% perform this task regularly.

A mouth care protocol was reportedly used on the ward by 69% of responders. Table 2 illustrates the reported percentage of patients requiring nursing assistance with oral hygiene maintenance. Ninety-four per cent of in-patient oral healthcare was carried out by nursing staff, auxiliary nursing staff or both.

**Table 2:** Reported percentage of patients requiring nursing assistance with oral hygiene maintenance.

	Total		A & E		Surgical		Critical Care		Medical		Bank	
	n	%	n	%	n	%	n	%	n	%	n	%
0–25%	23	19	1	20	18	42	0	0	4	10	0	0
26–50%	21	17	3	60	6	14	0	0	12	28	0	0
51–75%	16	13	0	0	6	14	1	4	9	21	0	0
76–100%	35	29	0	0	7	16	21	92	7	16	0	0
Variable	8	7	0	0	0	0	0	0	1	2	7	100
No Answer	15	12	1	20	5	12	0	0	9	21	0	0
Other	3	3	0	0	1	2	1	4	1	2	0	0

**Figure 1:**
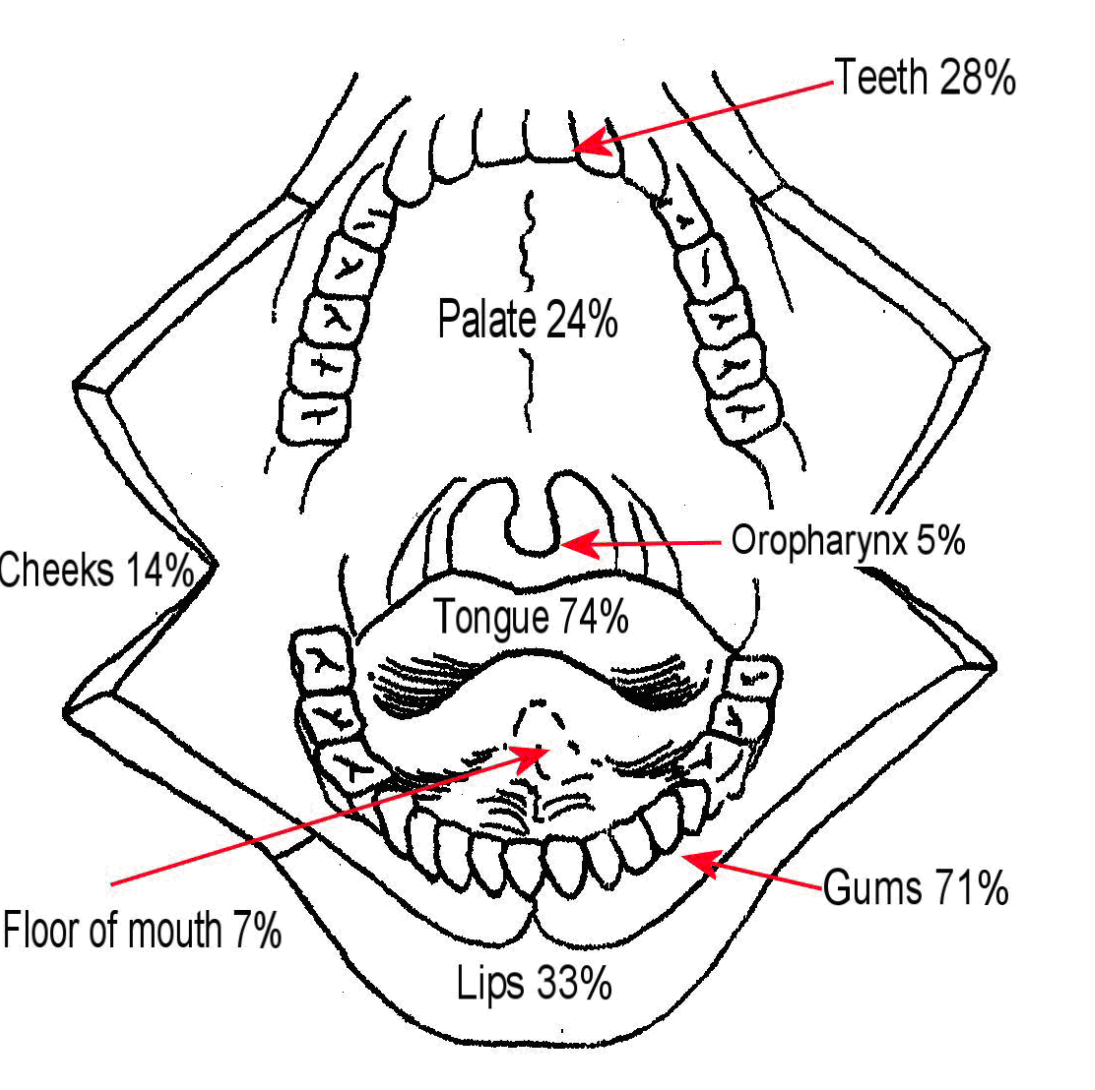
**Percentage of nurses identifying anatomical sites for identification during examination of the mouth**.

Figure 1 shows the anatomical sites for identification during examination of the mouth. Seventy four percent of nurses would examine the tongue but only 7% would examine the floor of mouth despite the potential for malignant change in this area.

**Figure 2:**
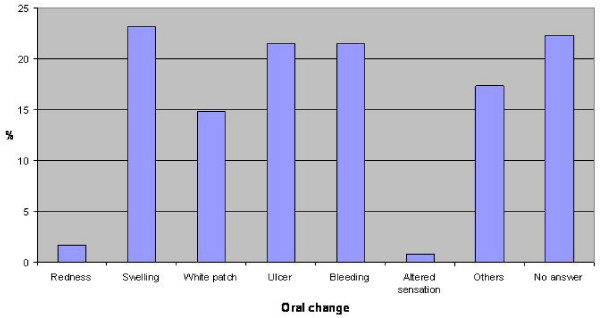
**Percentage of nurses identifying oral changes associated with oral cancer**.

Question 9 "What signs would make you suspicious of oral cancer?" was posed as an open question. Based on previous work by one of the authors [16,17] the answers shown in table 3 were accepted. Figure 2 shows the percentage of nurses identifying oral changes associated with oral cancer. In general less than 25% of nurses identified oral changes but in particular redness was identified poorly with only 2% of respondents identifying this oral change. There was no significant statistical difference in the mean number of oral changes identified by nurses from the different specialty groups or from different years from graduation (F = 1.936, p = 0.109 and F = 1.717, p = 0.167 respectively). There was no statistical difference in the mean number of oral changes identified between nurses who were regular dental attendees and those who were irregular dental attendees, t = 0.054, p = 0.478.

**Table 3:** Accepted oral changes associated with oral cancer.

Oral changes associated with Oral Cancer	
Ulceration	Exophytosis
Red patch (erythroplakia)	Fixation
White patch (leukoplakia)	Bleeding
Red/White patch (erythroleukoplakia)	Necrosis
Induration	Lymphadenopathy
Altered sensation	

**Figure 3:**
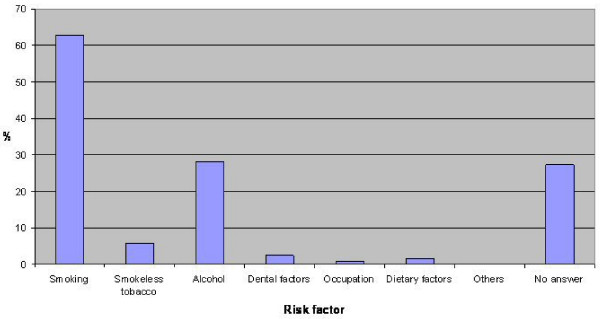
**Percentage of nurses identifying risk factors associated with oral cancer**.

Question 10 "What risk factors for oral cancer are you aware of?" was again posed as an open question. Based on previous work by one of the authors [16,17] the answers shown in table 4 were accepted. Figure 3 shows the percentage of nurses identifying risk factors associated with oral cancer. Smoking and alcohol use were the most commonly identified risk factors with 63% of nurses identifying smoking and 28% of nurses identifying alcohol as risk factors. The remaining risk factors were poorly identified. The mean number of risk factors identified by surgical (1.53) and critical care nurses (1.26) was greater than their accident and emergency (0.8), medical (0.83) and bank (0.28) colleagues F = 2.96, p = 0.023. The mean number of risk factors identified by nurses 1 to 3 years from graduation was significantly more than their colleagues F = 3.798, p = 0.012. There was no statistical difference in the mean number of risk factors identified by regular and irregular dental attendees, t = 0.790, p = 0.216.

**Table 4:** Accepted risk factors associated with oral cancer.

Risk factors for Oral Cancer	
Tobacco smoking	Viral factors
Smokeless tobacco use	Immunosuppression
Betel quid chewing	Chronic infection
Alcohol consumption	Occupation
Dietary factors	Pre-cancerous conditions/lesions
Dental factors	Previous history of oral cancer
UV light exposure	

Only five nurses reported regularly advising patients about risk factors for oral cancer. Four nurses reported that they advised patients about risk factors occasionally and 101 nurses reported that they did not advise patients regarding risk factors for oral cancer. This question was not answered by 11 nurses.

With regards to formal training on oral health care at nursing school and upon starting work after graduation: sixty-two per cent of nurses received formal training on oral health care at nursing school but only 35 per cent received any formal training on oral health care upon starting work. Auxiliary nurses were excluded from this question as they did not attend nursing school. Including auxiliary nurses 74 percent of nurses reported that they would like further training in oral health care.

## Discussion

In this changing era specialist nurses are expected to triage, diagnose and treat minor injuries. Recently prescribing, ordering investigations, meeting targets and providing out-of ours care are some aspects of the changing role of nursing staff. Nursing staff have the opportunity to be fundamental in changing the outcome of patients with undiagnosed oral cancer by recognising the early signs of the disease. The significance of their role in oral cancer detection has been previously outlined [9]. Interaction with nurses may allow high risk patients to increase their level of awareness and confidence to seek help when required.

The respondents to this questionnaire were nurses from a varied specialty background with the specialties grouped into surgical, medical, critical care, accident and emergency and bank nurses. The accident and emergency and bank nurse groups were small with only five and seven nurses in each group respectively. Results from these groups must be generalised with caution. Unfortunately demographic data regarding age and gender were not recorded as part of this pilot study. The time from graduation was recorded for those nurses attending nursing school which may provide a rough guide to the nursing experience as well as the age of the nurses questioned. Auxiliary nurses were also included in the study; although these nurses did not attend nursing school they did perform a significant proportion (up to 48 percent) of the oral health care of patients. Smoking and alcohol habits of the respondents were also not recorded, these could have affected responses; especially risk factors for oral cancer.

Over 80 percent of nurses agreed that it is important to examine a patient's mouth on admission; however, only 49 percent reported performing this task regularly. Performing an oral health check may depend on the nature and the projected length of admission. Sixty nine percent of nurses reported that their ward had a mouth care protocol. Oral examination has traditionally been the remit of doctors, dentists, and dental auxiliaries; however nurses who perform oral health care tasks should have the knowledge of basic surface anatomy of the oral cavity and be able to identify common pathological changes (red patches, white patches and ulcers) thus making examination of the mouth and opportunistic screening for oral cancer a realistic possibility.

This study did not investigate the anatomical knowledge of the nursing staff but did enquire as to which tissues were examined during an oral health check. The most common sites for oral cancer are the floor of mouth and ventral/lateral tongue. Seventy four percent of nurses reported examining the tongue; however only seven percent reported examining the floor of mouth despite the preponderance of oral cancers and pre-malignant changes in this area.

Signs of oral cancer were generally identified poorly. Ulceration is the most common presenting sign of oral cancer and less than 25 percent identified this. Up to 50 percent of red patches (erythroplakia) may have already progressed to invasive carcinoma yet a 'red patch' was identified as a sign of oral cancer by less than five percent of respondents. Interestingly, whether or not a nurse attended regular dental care did not seem to have an impact on clinical sign identification.

Over 60 percent of nursing staff questioned identified smoking as a risk factor for oral cancer with just less than 30 percent identifying alcohol. Other risk factors were identified less well. This data parallels work undertaken by one of the authors on medical and dental students' awareness of oral cavity carcinoma [16]. The surgical and intensive care nursing staff scored significantly higher than their colleagues; these groups included staff from wards where Maxillofacial and Otorhinolaryngology patients' are cared for. Nursing staff within one to three years of qualification identified a significantly greater mean number of risk factors than their more senior counterparts. This highlights a need for continuing medical education, supported by Wardh *et al*., who used a questionnaire to test nursing and auxiliary nursing staff on oral healthcare. The two groups underwent a four hour teaching programme and repeated the questionnaire two years later [18]. It was apparent from these results that specific knowledge was not retained thus demonstrating the key to continuous use of the new skill and this aspect should be covered in undergraduate and postgraduate teaching for reinforcement.

Interestingly only five nurses reported advising patients regularly about risk factors for oral cancer. Delivering advice on oral cancer may directly depend on the nature of the hospital admission. Also hospital nurses may not feel that it is their role to deliver advice regarding cancer risk without further training. Nurse practitioners, i.e. nurses educated at graduate level, have the skill-set to conduct physical examinations, assess risk factors and screen patients for disease. Unfortunately this study did not identify which nurses were educated to this level but should be investigated in future studies. Lack of awareness of oral cancer risk and clinical signs may also prohibit nurses from delivering preventive advice. Our results demonstrated only 61 percent of nursing staff in this study received oral healthcare training at nursing school with 34 percent receiving postgraduate education. It was not assessed whether this was related purely to oral hygiene maintenance or encompassed oral diseases. Seventy-four percent of staff requested further training to improve patient care.

The results of this study indicate that whilst there is desire to increase patient total care, teaching is required to enhance awareness of oral cancer risk factors and signs. Adams (1996) through a questionnaire targeted nurses on acute elderly care and general medical wards on their knowledge of oral healthcare [19]. The study was not focused at cancer detection but to oral hygiene issues. The study highlighted, in keeping with our work, that nurses thought oral care was an important part of hospital care. The paper went onto surmise that although nurses do receive oral healthcare training as part of their curriculum, this is most often not taught by a specialist in that area. It is therefore appropriate that medical or dental staff that have specialist interests in this area; oral medicine specialists, oral and maxillofacial surgeons, otorhinolaryngologists, plastic surgeons, specialist nurses in these areas, specialist oncology nurses, dentists, dental hygienists, dental therapists or oral health educators could in future train nursing staff on oral healthcare including oral cancer awareness. It is essential to include adequate training in the nursing curriculum as clinical observation and oral examination by nurses may prove effective in improving survival rates for oral cancer [20]. Oral health checks were performed by nursing staff on patients with haematological malignancies by Andersson and colleagues [21]. The nurses' evaluation of the oral cavity was comparable to a dental hygienist. The nurses had been trained for two hours prior to implementation of this and on average the examination took five minutes and was undertaken every day. Although this study was not investigating oral cancer detection, but oral and oral related issues in general, it can be inferred that teaching with regular use of the new skills could allow for a 'screening' process which is effective without adding too much extra burden to already hard working nursing staff.

Our data is consistent with research published elsewhere in that targeted education is needed to prepare oral health providers to undertake oral cancer prevention activities [22]. This study highlighted weakness in the training of nurses similar to those reported previously involving the training of medical and dental students [16]. In addition the results in this study reflect those obtained in a previous study involving general practitioners that identified the need for improved education [17]. All of these studies highlighted a need to emphasize the role of alcohol as well as tobacco as a risk factor; and to emphasize the importance of early oral mucosal changes in particular ulcerative lesions and red and white patches. The government has already stated that a key area is earlier detection of cancer which affords quicker treatment that can save lives. The department of health, cancer reform strategy (2007); states that non-medical screening for oral disease may be the focus of the future as individuals at risk from oral cancer are unlikely to have a general dental practitioner [23]. The possibility of community nurse clinics or pharmacy advice on oral ulceration would allow increased awareness in the community. There will always be patients who avoid such initiatives and therefore, a hospital oral health check would hopefully pick-up those individuals who had been admitted to hospital. Whilst this is not a formal screening programme the ability to 'screen' individuals especially those at risk who are admitted to hospital would allow detection of tumours at an earlier stage.

## Conclusion

An oral health check upon admission to hospital provides an opportunity for nurses to 'screen' for oral diseases including oral cancer and allows nurses a greater role in total patient care. Nursing staff regarded an oral health check as important; however, nurses' awareness of oral cancer risk factors and clinical signs was poor. This study highlights a need for improved education of nurses on oral cancer to make the oral health check on admission viable for oral cancer screening.

## Competing interests

The authors have no financial and personal relationships with other people, or organisations, that could inappropriately influence (bias) their work, all within 3 years of beginning the work submitted.

## Authors' contributions

LMC conceived the study. VPK, SJ and LMC developed the questionnaire. VPK and LMC delivered the questionnaires. ATH, AK, SJ and LMC prepared the manuscript. ATH and LMC edited the discussion and prepared the final draft of the paper. All authors read and approved the final draft of the manuscript.

## Appendix A

HULL AND EAST YORKSHIRE HOSPITALS NHS TRUST

DEPARTMENT OF ORAL AND MAXILLOFACIAL SURGERY

ORAL HEALTH CARE AWARENESS AMONGST NURSES

1) Do you carry out an oral health check on a patient's admission?

            a) Yes         b) No

2) Do you think it is important to examine a patient's mouth on admission?

            a) Yes         b) No

3) Does your ward have a mouth care protocol?

            a) Yes         b) No

4) What percentage of patients on your ward require nursing assistance with oral hygiene maintenance?_(Please specify)_

5) Who carries out oral care on your ward?_(Please specify)_

6) Do you have any practical difficulties in carrying out regular oral health care for patients on your ward?

            a) Yes_(Please specify)_         b) No

7) Which tissues do you examine when assessing oral health?_(Please specify)_

8) What signs do you think would make you suspicious of an oral disease?

9) What signs would make you suspicious of an oral cancer?

10) What risk factors for oral cancer are you aware of?

11) Do you regularly advise patients about risk factors for oral cancer?

12) What training did you receive with regards to oral health care at nursing school?

13) What training have you had on oral health care since starting work on your ward?

14) Would you like further training in oral health care?

            a) Yes_(Please specify)_         b) No

Thank you for your time and energy!

## Pre-publication history

The pre-publication history for this paper can be accessed here:


